# Predictive Role of Leptin Receptor (Ob-R) Overexpression in Patients with Early Breast Cancer Receiving Neoadjuvant Systemic Treatment

**DOI:** 10.3390/cancers13133269

**Published:** 2021-06-29

**Authors:** Laura García-Estévez, Isabel Calvo, Silvia Pérez, Isabel Gallegos, Eva Díaz, Miguel Sampayo-Cordero, Sara S Oltra, Gema Moreno-Bueno

**Affiliations:** 1 Foundation MD Anderson International, C/Gómez Hemans 2, 28033 Madrid, Spain; icalvo@mdanderson.es (I.C.); sperez@mdanderson.es (S.P.); mgallegos@mdanderson.es (I.G.); eva.diaz@fundacionmdanderson.es (E.D.); soltra@iib.uam.es (S.S.O.); gmoreno@iib.uam.es (G.M.-B.); 2 Centro de Investigación Biomédica en Red de Cáncer (CIBERONC), 28029 Madrid, Spain; 3 Medica Scientia Innovation Research (MedSIR), 08018 Barcelona, Spain; miguel.sampayo@optimapharm.eu; 4 Optimapharm, Biostatistics Department, Parc Bit Edifici Disset A2, 07121 Palma de Mallorca, Spain; 5 Biochemistry Department, Instituto de Investigaciones Biomédoicas ‘Alberto Sols’ (CSIC-UAM), Universidad Autónoma de Madrid (UAM), IdiPaz, 28029 Madrid, Spain

**Keywords:** leptin receptor, Ob-R, breast cancer, predictive biomarker

## Abstract

**Simple Summary:**

Obesity and overweight, considered the pandemic disease in the 21st century, is highly related to breast cancer. Leptin receptor (Ob-R) and its ligand leptin display an important role in driving this connection. Nowadays, translational cancer research is mainly focused on the identification of new biomarkers able to discriminate those patients deriving greater efficacy from a given therapy. In this regard, our study examines the role of Ob-R, namely, the correlation between Ob-R and pathological complete Response (pCR) in early breast cancer patients receiving neoadjuvant systemic therapy. Here, we decoded the correlation of Ob-R with certain clinical features such as breast cancer subtype, age, body mass index (BMI), menopausal status, and mammogram breast density. The study provides further support for the potential value of Ob-R for the first time as a possible role for predicting pCR. Moreover, we would like to highlight the importance of Ob-R as independent predictive factors for pCR in breast cancer patients.

**Abstract:**

The primary aim of this retrospective study was to investigate the correlation between the immunohistochemical expression of Ob-R (leptin receptor) with pCR (pathological complete response) in early breast cancer patients receiving neoadjuvant systemic treatment (NST). A total of 100 women with breast cancer receiving NST (2017–2020) followed by surgical resection were retrospectively obtained. Demographic parameters and clinicopathological factors (e.g., treatment modalities, immunohistochemistry (IHC), and cancer subtype) were obtained from the patient’s clinical records. In the analyzed breast cancer cohort, high expression of Ob-R was found in 52% of tumors and there was a significantly higher incidence in the HER2+ and TNBC subgroups. Overall, a significantly greater percentage of patients with Ob-R positive tumors achieved pCR compared with Ob-R negative patients (57.7% vs. 27.1%; *p* = 0.002). This result was observed in most breast cancer subtypes. In patients with HER2+ breast cancer, there was no difference in Ob-R expression in relation to the HR status. Ob-R cell positivity was significantly higher in younger breast cancer patients (*p* = 0.008), those who were premenopausal (*p* = 0.011), and in those with a BMI > 25 kg/m^2^ (*p* = 0.019). A significantly greater percentage of early breast cancer patients with Ob-R positive tumors achieved pCR compared with Ob-R negative patients. Furthermore, breast cancer patients with positive Ob-R expression were significantly younger than those with negative Ob-R expression. This association was not explained by differences in BMI between young and old patients.

## 1. Introduction

Neoadjuvant systemic treatment (NST) is one of the most common strategies for treating early breast cancer. In certain cancer subtypes such as triple negative (TNBC), HER2 positive, or high-risk luminal-like HER2 negative, NST is the standard of care especially for tumors larger than 2 cm and/or affected axillary lymph nodes [1]. An advantage of NST is that it enables monitoring of treatment effect using biomarkers predictive of in vivo tumor sensitivity and potential pathological response. The pCR has been a controversial endpoint since complete disappearance of the tumor does not necessarily always translate into better survival [2]. However, it still continues to be the primary objective endpoint in the majority of clinical trials in the neoadjuvant setting due to its correlation with long-term patient outcomes and, additionally, adjuvant therapy choice may differ based on the pCR status [3].

As an increasing number of breast cancer patients are treated with NST, identification of biomarkers to predict the probability of pCR in individual cases is a high priority, since these could provide insights into breast cancer pathogenesis and support personalized cancer management. Today, there is no reliable, clinically validated, method for predicting pathological responders from non-responders. Established molecular biomarkers currently identified include those related to receptor status such as estrogen receptors (ER), progesterone receptor (PR), and human epidermal growth factor receptor 2 (HER2); the proliferation marker, Ki67, and other markers including the genomic grade index and tumor-infiltrating lymphocytes [4]. While we are making steady improvements with biomarkers in breast cancer, there is still an unmet need for more accurate predictors of factors that impact pCR, especially in the neoadjuvant setting.

Obesity represents an established risk factor for breast cancer, and potential biological mechanisms underlying this relationship include insulin resistance and abnormalities in the insulin-like growth factor-1 (IGF-1) axis, sex hormones biosynthesis, subclinical chronic inflammation, and alterations in adipokine pathophysiology [5]. Through alterations in these pathways, obesity can influence cancer cell survival, metastasis, angiogenesis, apoptosis, and the cancer microenvironment, and thus increase the risk of breast cancer development [6,7]. The incidence of breast cancer in postmenopausal woman has been shown to rise as BMI increases, and adipokines released from the increased adipose tissue mass, in particular the adipocytokine leptin, are considered to be key drivers of breast cancer tumorigenesis [8,9,10]. There is a strong positive correlation between leptin mRNA and protein levels in adipose tissue and circulating leptin levels [11,12,13]. Functionally, leptin exerts its effects by binding to its receptor (Ob-R) resulting in the activation of several signaling pathways including the Janus kinase/signal transducer and activator of transcription (JAK/STAT), mitogen-activated protein kinase (MAPK), and phosphatidylinositol-3-kinase (PI3K) pathways, which ultimately promote cell proliferation [14]. Leptin signaling through the leptin receptor also activates angiogenesis [15] and might be an important mediator between the tumor and its microenvironment [16]. Therefore, leptin through binding to its receptor is involved in the tumorigenesis of breast cancer.

Moreover, a number of studies have highlighted a link between leptin and the leptin receptor (Ob-R) overexpression with more aggressive breast cancer as evidenced by distant metastases, lymph node metastases, and increased tumor size [10,17]. An overview of systematic reviews explored the association between circulating leptin levels and risk of breast cancer, and all of them showed an increased breast cancer risk with higher leptin levels in postmenopausal women, and the majority also found the same association in all women [5]. Therefore, there might be a correlation between serum leptin levels, leptin, and Ob-R expression in breast cancer patients which correspond with increased cancer risk, although there are currently no definitive data on this topic.

The primary objective of this study was to investigate the correlation between the immunohistochemical expression of Ob-R with pCR in early breast cancer patients receiving neoadjuvant treatment. A secondary aim was to analyze the correlation of Ob-R with breast cancer subtypes and others clinical variables such as age, menopausal status, BMI, and mammographic breast density (MBD).

## 2. Material and Methods

### 2.1. Study Design

This was a retrospective study of breast cancer patients included on the computerized database system at MD Anderson International Foundation Biobank (record number B.0000745, ISCIII National Biobank Record), Madrid, Spain, between 2017 and 2020. The study was conducted in accordance with the Declaration of Helsinki, and all patients provided written informed consent for the analysis of tumor biopsies for biomarker assessment. This study was approved by the Hospital Ramón y Cajal Ethics Committee (30 November 2020, Acta 402).

### 2.2. Patient Population and Treatment

A total of 100 women with breast cancer treated with NST followed by surgical resection were included in this real-world analysis in everyday clinical practice. Neoadjuvant combination treatment comprised standard chemotherapy plus anthracyclines and cyclophosphamide every 21 days for 4 cycles, followed by weekly paclitaxel for 12 weeks. Carboplatin (AUC 2 for 4 cycles) was added in patients with TNBC. HER2 positive cases also received anti-HER2 monoclonal antibody (mAb) therapy with pertuzumab plus trastuzumab. Surgery was performed within 4–6 weeks of the last chemotherapy dose.

### 2.3. Procedures

Demographic parameters including age, menopausal status, and weight/height (to calculate BMI) and clinicopathological factors (e.g., treatment modalities, immunohistochemistry (IHC), and tumor staging) were obtained following a review of each patient’s clinical records. MBD assessment was performed during a mammography examination at the time of initial diagnosis. The American College of Radiology classification was applied and described 4 types of breast density: (A) almost entirely fatty; (B) scattered fibroglandular tissue; (C) heterogeneously dense fibroglandular tissue; and (D) extremely dense fibroglandular tissue [18].

To evaluate the molecular subtype classification, IHC analysis of ER, PR, and Ki-67 was performed for samples available pre-NST and using established criteria. HER2 expression was determined according to the criteria of American Society of Clinical Oncology (ASCO)/College of American Pathologist (CAP) guidelines [19]. Tumors with scores 2+ were further tested by fluorescence in situ hybridization (FISH). IHC for Ki-67 using monoclonal antibody (clone MIB-1, Dako (Jena, Germany), dilution 1:300) was scored using an automated image analysis system (i-Solution DT made by IMT i-Solution Inc., Riverton, UT, USA), as the percentage of positively stained tumor cells within the highest proliferative area (hot spot). The level of Ki-67 expression was classified as high versus low with a cut-off point of 20% [20,21]. Based on the IHC results, tumors were categorized using the St Gallen criteria, as luminal A (ER/PR+, HER2−, and low Ki-67), HER2− luminal B (ER+, HER2−, and either high Ki-67 or PR−), HER2+ luminal B (ER+ and HER2+), HER2+ (ER− and HER2+), or triple negative (ER/PR− and HER2−) subtypes [22].

### 2.4. Ob-R Expression by Immunohistochemical Analysis

Ob-R expression was routinely measured using the BOND RX Research Platform (Leica Biosystems, Wetzlar, Germany) following the manufacturer’s instructions. Briefly, tissue samples were obtained from 100 breast cancer patients at the time of diagnosis, and each sample was fixed in 10% buffered formalin before being embedded in a paraffin block. IHC analysis was performed using 3 µm sections of breast tissue which were deparaffinized and immunostained with BOND RX^m^ autostainer (Leica Biosystems) using BOND Epitope Retrieval Solutions and the BOND Polymer Refine Detection kit (Leica Biosystems). Moreover, Ob-R expression was tested by immunohistochemical staining using goat polyclonal antibodies against Ob-R (M-18, 1:30 dilution; Santa Cruz Biotechnology, Dallas, TX, USA). The expression of Ob-R was analyzed by light microscopy in 10 different section fields, and the mean percentage of tumor cells displaying positive staining was scored. A semiquantitative scoring of each marker, based on staining intensity and the percentage of stained tumor cells was applied by two independent observers, blinded to the clinical data. The expression of Ob-R in cancer samples was classified using a four-point scale: 0, <10% positive cells; 1+, 10% to 50% positive cells with weak staining; 2+, >50% positive cells with weak staining; 3+, >50% positive cells with strong staining [23].

### 2.5. Endpoints and Definitions

The primary objective of this study was to investigate potential correlations between immunohistochemical expression of Ob-R with pCR in early breast cancer patients receiving neoadjuvant therapy. Leptin receptor expression was categorized as either ‘positive’ or ‘negative/low positive’. Ob-R cell positivity was defined as >50% positive cells with weak staining (2+) or >50% positive cells with strong staining (3+) while Ob-R cell negativity/low positivity was defined as <10% positive cells (0) or 10% to 50% of positive cells with weak staining (1+) [23]. A previous study suggested clinical differences between patients with Ob-R ≤ 50% and >50% [24]. The pCR endpoint was defined as disappearance of invasive cancer in the breast and axillae (pT0/is ypN0) based on histopathologic analysis of surgical specimens [2].

Secondary objectives included (1) the association of Ob-R expression with different cancer subtypes (HER2+/HR−, HER2+/HR+, triple negative, luminal A, and luminal B); (2) the association between Ob-R cell positivity and pCR rate with cancer subtype; and (3) the association between Ob-R expression with patient characteristics.

### 2.6. Statistical Methods

We planned a sample size of 100 patients to provide 80% power with a significance level of 5% (two-sided) to detect an absolute difference in pCR in the breast and axillae of 30% between patients with and without Ob-R cell overexpression [25]. We considered a 10% drop-out rate. The primary outcome and all secondary outcomes were analyzed in the evaluable population which included patients evaluated for pCR and Ob-R status.

The primary analysis was conducted with univariate and multivariate logistic regression models. The *p*-value and the 95% confidence interval for the odds ratio (OR) were calculated using the Wald test. In the full model, the multivariate analysis included all prognostic factors reported in Table 1. Predictors and confounders included in the final model were selected at a *p*-value of <0.1 or change in OR > 10% compared to the full model. The association between pCR or Ob-R expression with cancer subtypes and patient characteristics was analyzed using the same statistical approach described for the primary analysis. To compare median age, height, and weight between patients with and without pCR, we used a U-Mann Whitney’s test. The significance level was set to a two-sided α of 0.05. We used R version 4.0.2 for all statistical analyses.

## 3. Results

### 3.1. Patient Characteristics

The demographic and clinicopathologic characteristics of the 100 patients included in this analysis are shown in Table 1. The median age of patients was 46 (range 29–81) years, 35% were postmenopausal, and 32% had a BMI ≥ 25 kg/m^2^ (within this group 22% were overweight and 10% were obese).

**Figure 1:**
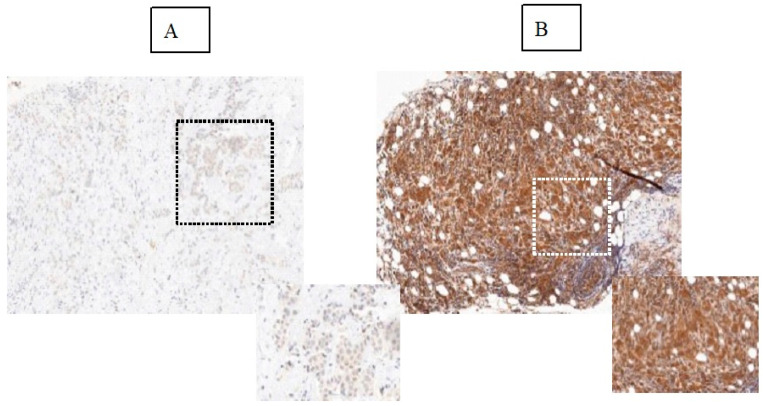
Immunohistochemical detection of Ob-R expression including examples of breast tumors with low (**A**) and high expression of Ob-R (**B**) at ×20 magnification. Highlighted insets show magnification (×40) areas. Ob-R expression classification as described by Garofalo et al. 2006 [23].

Key histopathological data for this group of patients are presented in Table 1 and 80% had MBD type C or D; almost equal numbers of patients had breast cancer subtypes HER2+ (35%), triple negative (31%), or luminal (34%); 96% had histological Grade 2 or 3; 83% had a T (tumor) size of 1–2; 97% had a nodal status of N0 (50%) or N1 (47%); and 52% of patients had an Ob-R positivity of >50% (+2, +3) vs. 48% of patients with an Ob-R positivity of ≤50% (0, +1) (Figure 1).

### 3.2. Ob-R Overexpression Is a Significant Predictive Factor for pCR in the Global Breast Cancer Population

In our study, 43 patients achieved a pCR and 57 presented with residual disease at definitive surgery (Table 1). As expected, luminal tumors showed significantly lower percentage of pCR (17.6%) than HER2 (57.1%) and TNBC (54.8%).

**Figure 2:**
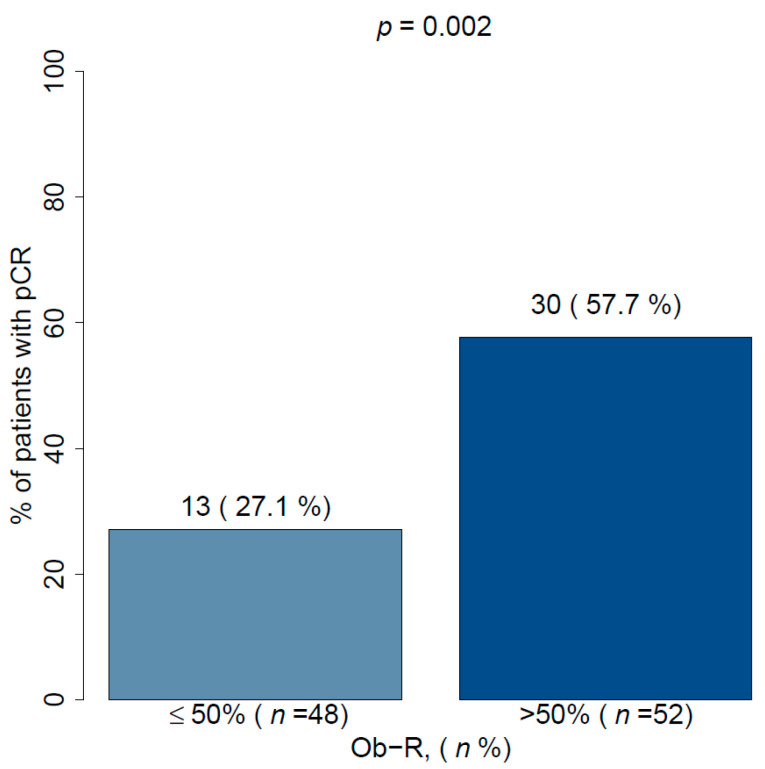
Pathological complete response in accordance with Ob-R cell overexpression (>50%). Ob-R: Leptin receptor, pCR: pathological complete response.

**Figure 3:**
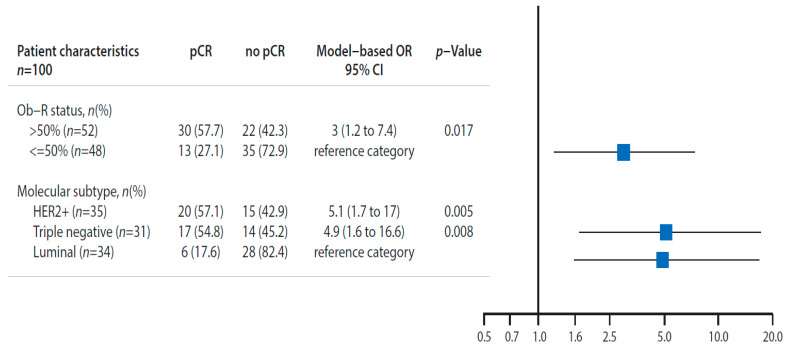
Pathological complete response in accordance with Ob-R overexpression (>50%). Multivariate logistic regression model analysis. The multivariate analysis has included all prognostic factors reported in Table 1 in the full model. Factors included in the final model have been selected at a *p*-value of <0.1 or change in OR > 10% compared to the full model. The factors selected in final model are presented. The pCR rate was significantly greater in patients with Ob-R overexpression (>50%) than patients without Ob-R overexpression (≤50%). In addition the HER2+ and triple negative patients has a pCR significantly greater than luminal patients. 95% CI: 95% confidence interval; Ob-R: Leptin receptor; pCR: pathological complete response; HER2: Human epidermal growth factor receptor 2.

With regards to the primary objective, Ob-R positive tumors (+2 and +3 score) showed significantly greater percentage of pCR than Ob-R negative patients (+1 and 0 score) (57.7% vs. 27.1%; *p* = 0.002) in univariate and multivariate analyses (*p* = 0.017) ( Figure 2; Figure 3, and Table 1).

### 3.3. HER2 and TNBC Breast Cancer Show High Levels of Ob-R

**Figure 4:**
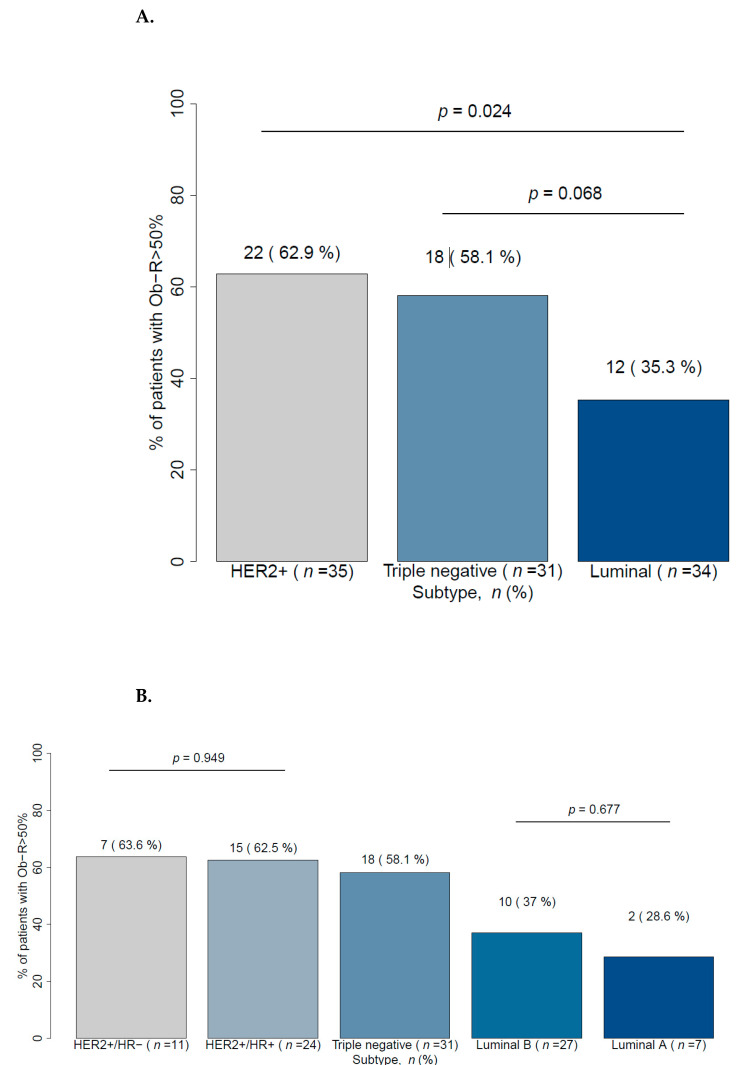
Ob-R cell overexpression (>50%) in accordance with cancer classification in (**A**) three molecular subtypes and (**B**) five molecular subtypes. HER2: Human epidermal growth factor receptor 2; HR: Hormonal receptor; Ob-R: Leptin receptor.

We investigated if there were any differences in Ob-R levels with respect to breast cancer subtype and recorded Ob-R cell positivity (+2, +3) rates in patients with HER2+, TNBC, and luminal breast cancer of 62.9%, 58.1%, and 35.3%, respectively (Figure 4A). Multivariate analyses showed that there was a different distribution of Ob-R overexpression according to cancer subtype; for example, patients with HER2 or TNBC breast cancer had significantly higher Ob-R cell positivity than patients with luminal subtypes (Table 2). With regard to luminal breast cancer, the expression of Ob-R was similar between luminal subgroups A and B (37% and 28.6%, respectively) (Figure 4B). Likewise, within the group of HER2+ patients (HER2/HR− and HER2/HR+) there was no significant difference with respect to Ob-R overexpression (63.6% and 62.5%, respectively) (Figure 4B).

### 3.4. The Correlation between Ob-R Overexpression and pCR Rate Depends on the Breast Cancer Subtype

**Figure 5:**
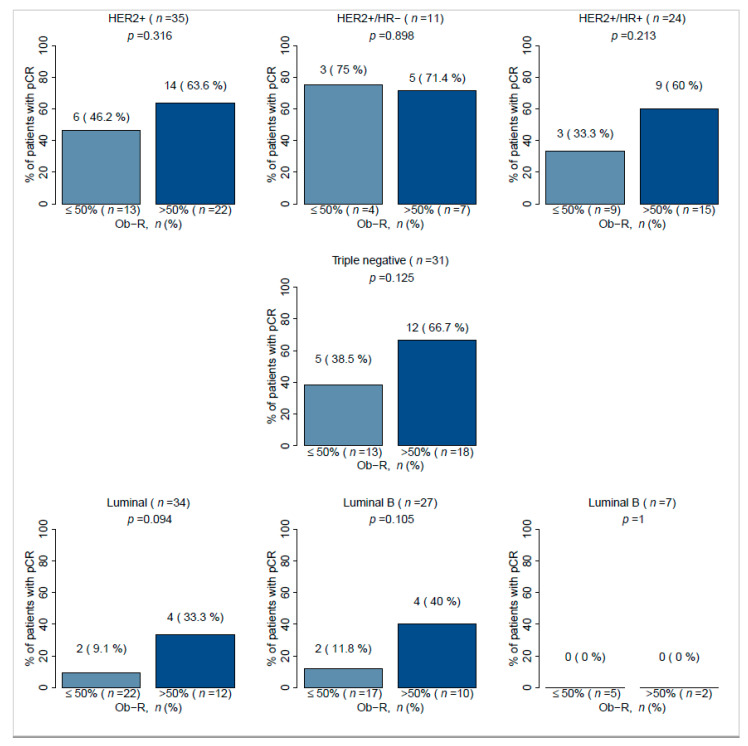
Association between Ob-R cell positivity and pCR in accordance to breast cancer molecular subtypes. HER2: Human epidermal growth factor receptor 2, HR: Hormonal receptor, Ob-R: leptin receptor, pCR: Pathological complete response.

Investigation of the correlation between pCR and Ob-R overexpression according to breast cancer subtype showed a non-significant trend among the three subtypes HER2+, TNBC, and luminal tumors. Within each subtype, patients with positive Ob-R tumors appear to be more likely to achieve a pCR than patients with negative Ob-R tumors (Figure 5). Within the subgroup of patients with the HER2+/HR− tumors (n = 11) there was no difference in pCR between Ob-R positive tumors (71.4%) versus Ob-R negative tumors (75%). In contrast, in the subgroup of HER2+/HR+ patients (n = 24), there was a marked difference in pCR between Ob-R positive and negative subgroups (60% vs. 33.3%), but the result did not achieve statistical significance (*p* = 0.213). Similarly, in tumors classified as luminal B (n = 27), the pCR response was much higher in Ob-R positive tumors compared with Ob-R negative tumors, but the difference again did not achieve statistical significance (40% vs. 11.8%, *p* = 0.105). The same non-significant trend was observed in TNBC patients when comparing Ob-R positive and negative tumors (66.7% vs. 38.5%, *p* = 0.125). No luminal A tumors achieved a pCR.

### 3.5. Ob-R Overexpression Is Highly Correlated to Age and Menopausal Status in Breast Cancer

Age is considered prognostic factor in breast cancer [26,27]. We, therefore, investigated whether OB-R expression was different with respect to age in our cohort of breast cancer patients. Breast cancer patients with positive Ob-R expression (+2, +3) were significantly younger that those with negative Ob-R tumors [median (range): 44 (29–73) years vs. 51.5 (33–81) years; *p* = 0.008]. Furthermore, this association was not explained by differences in BMI or breast cancer subtype between young and old patients as observed in the multivariate logistic regression model (Table 2).

Similarly, Ob-R overexpression was noted in significantly more premenopausal patients compared with postmenopausal patients (61.5% vs. 34.3%; *p* = 0.011). Again, this association was not explained by differences in BMI between premenopausal and postmenopausal patients or to the subtype of cancer included in the multivariate logistic regression model (Table 2).

### 3.6. Ob-R Overexpression Is Significantly Associated with BMI in Breast Cancer Patients

Leptin, the ligand of Ob-R, is directly related to the adipose tissue mass and hence, BMI. Obese people present with higher leptin levels than lean people [28]. In this regard, we further analyzed the association between Ob-R and BMI. Overall, 68 patients were underweight (BMI < 18.5 kg/m^2^) or normal weight (18.5 to 24.9 kg/m^2^), whereas 22 patients were overweight (25 to 29.9 kg/m^2^) and 10 were obese (≥30 kg/m^2^). A greater percentage of overweight and obese patients had highly positive Ob-R expression compared with normal or underweight patients (62.5% vs. 47.1%). This difference was statistically significant (*p* = 0.019) in multivariate analysis with other relevant patient characteristics such as age and menopausal status included in the model (Table 2).

### 3.7. Ob-R Overexpression Showed Not Association with Mammographic Breast Density (MBD)

MBD is defined by the proportion of glandular to fatty tissue in the breast. Because of the relationship between leptin expression and fat tissue, we evaluated whether individuals with greater fatter content (MBD types A and B) had higher expression of Ob-R compared with patients with denser breast tissue (MBD types C and D). Overall, 17 patients presented with MBD types A or B and 83 with type C or D. There was no significant difference between these two groups with respect to Ob-R overexpression (41.2% vs. 53.8%; *p* = 0.349).

## 4. Discussion

Leptin is a multifunctional neuroendocrine peptide hormone, mainly secreted by differentiated adipocytes, and it is involved in food intake, satiety, energy expenditure, and reproduction [29]. Plasma leptin levels are proportional to fat mass and increase as body weight rises [30]. Leptin exerts its effects by binding to Ob-R, and this receptor exists in six isoforms resulting from different alternative splicing of the gene: four short isoforms (Ob-Ra, Ob-Rc, Ob-Rd, and Ob-Rf), one long isoform Ob-Rb, and the soluble Ob-Re isoform whose main function is to control serum leptin levels. Ob-Rb has an extracellular domain with leptin binding site, transmembrane domain, and long intracellular domain which enables activation of several intracellular pathways and it can activate several signaling pathways like JAK2/STAT as well as other downstream cascades such as PI3K/AKT and MAPK [31].

Ob-R is very low or negatively expressed in epithelial cells from normal mammary gland tissue, whereas expression has been reported to be high in breast cancer cells [23]. It has been hypothesized that the expression of Ob-R is induced during the tumorigenesis of breast cancer [14]. Furthermore, the expression of leptin and its receptor Ob-R were positively correlated, suggesting that leptin acts on mammary tumor cells via an autocrine pathway [17,32,33].

In the current study we evaluated the role of Ob-R expression and its correlation with pCR, breast cancer subtypes, and clinicopathological variables in a breast cancer population. In the overall population high expression of Ob-R was found in 52% of tumors and there was a significantly higher incidence in the HER2+ and TNBC subgroups. In patients with HER2+ breast cancer there was no difference in Ob-R expression in the HR+ and HR− subtypes. Ob-R cell positivity was significantly higher in younger breast cancer patients (*p* = 0.008), those who were premenopausal (*p* = 0.011), and in overweight/obese patients (*p* = 0.019).

These results are consistent with data from other studies where leptin and Ob-R are overexpressed and closely correlated with breast cancer tumorigenesis [17,23,32,33]. However, the definition of Ob-R positivity was not standardized and varied considerably in these studies. We used the same classification as Garofalo and collaborators [23].

In our study a significantly greater percentage of patients with Ob-R positive tumors achieved a pCR compared with Ob-R negative patients (57.7% vs. 27.1%; *p* = 0.002). This result has been confirmed in a multivariate logistic regression model. Improved pCR was documented for HER2+/HR+, TNBC, and luminal B subtypes of breast cancer with high Ob-R cell positivity, but these subgroups contained relatively small numbers of patients and did not achieve statistical significance. Pathological response rates according to Ob-R overexpression highlighted very little difference in patients with positive or negative Ob-R expression in the HER2+/HR− (71.4% vs. 75%) and the Luminal A group (0% vs. 0%). In contrast, in the HER2+/HR+ group, fewer patients in the negative Ob-R expression group achieved pCR (33.3%) compared with the positive Ob-R expression group (60% of patients). These data would need to be validated with larger series. If confirmed, Ob-R might be an interesting predictive factor to be used in a population with a lower rate of pCR compared to HER2+/HR− group. A similar result was observed for Luminal B patients with negative Ob-R expression (pCR: 11.8%) compared with positive Ob-R expression (pCR: 40%).

Why the presence of Ob-R is a predictor of tumor response in early breast cancer patients receiving neoadjuvant treatment is unknown. It is noteworthy, however, that it is more frequently observed in more aggressive breast cancer subtypes, and also those more sensitive to neoadjuvant chemotherapy such as TN and HER2+ tumors. One possible explanation is that Ob-R overexpression in tumor cells might activate the leptin-Ob-R-JAK2/STAT3 axis as well as other pathways such as PI3K/AKT and MAPK producing a proliferative stage through which is more sensitive to chemotherapy. The precise mechanisms remain unknown, but they constitute an interesting area for future research.

The clinical association of leptin or Ob-R with cancer patient outcome have been explored in different cancers. In general, the expression of leptin and its receptor were found to be associated with poor prognosis in the majority of cancers studied; however, negative correlation with cancer progression has also been reported [34]. Furthermore, a limited analysis by Ishikawa et al. found that patients with Ob-R positive tumors and greater expression of leptin were associated with cancer recurrence in distant organs and lower rates of survival after seven years compared with patients with Ob-R negative tumors and low expression of leptin [17].

With regard to association of Ob-R and breast cancer subtypes, our study suggests that there is a significant association with HER2+ and TNBC. Garofalo and colleagues found no significant association with the expression of ERα, ERβ, or Ki-67 [23]. However, these results contrast with results presented by other authors who reported that Ob-R expression was higher for non-TN breast cancers (*p* = 0.045) and breast cancers with a high Ki-67 labeling index [33]. Moreover, other studies have shown that Ob-R might be an independent biomarker for different types of breast cancer, not correlating with ER/PgR or HER2 status [35,36]. In one of these studies, leptin and Ob-R were found in both HER2+ and HER2- tumors suggesting that the two systems are controlled by different mechanisms [35].

In our study, the association of Ob-R with HER2+ and TN tumors is difficult to explain, given the contradictory data in the literature. However, without estrogen signaling, it is attractive to speculate with the concept that tumor progression may be driven by other signaling factors such as fibroblast growth factor receptor-1 (FGFR-1), insulin growth factor 1 (IGF-1), or Ob-R [37,38].

Plasma leptin levels are proportional to fat mass and its increase as body weight rises [27]. It is possible that a similar situation occurred with Ob-R. In our study, overweight and obese patients had a higher likelihood of Ob-R positive tumors (+2, +3) compared with underweight and normal weight patients.

There appear to be no previous data correlating Ob-R with age and menopausal status, and the significant association in favor of young and premenopausal patients cannot be explained by higher BMI or specific breast cancer subtypes and is worthy of future study.

It is important to acknowledge the limitations of the current exploratory study. Firstly, retrospective studies are considered to provide weaker levels of evidence compared with prospective studies since they increase the potential for selection and information bias. However, they do reflect everyday clinical practice and results can be obtained more rapidly. Another weakness of the study is the relatively small number of patients included and this made it difficult to identify statistically significant trends in the small groups analyzed.

## 5. Conclusions

In the overall population, high expression of Ob-R was found in 52% of tumors and a significantly greater percentage of patients with Ob-R positive tumors achieved a pCR compared with Ob-R negative patients. We also observed a trend among the three breast cancer subtypes (HER2+, TNBC, and luminal tumors) such that patients with positive Ob-R tumors appeared to be more likely to achieve a pCR than patients with negative Ob-R tumors. Furthermore, breast cancer patients with positive Ob-R expression were significantly younger than those with negative Ob-R tumors (median 44 years vs. 51.5 years; *p* = 0.008). This association was not explained by differences in BMI between young and old patients.

## Figures and Tables

**Table 1:** Demographic and patient characteristics of total population and pCR groups.

Patient Characteristics	All Patients * (*n* = 100)	With pCR * (*n* = 43)	Without pCR * (*n* = 57)	*p*-Value
Age, years				
Median (range)	46 (29–81)	46 (29–81)	47 (33–73)	0.427
<50 years	59 (59)	28 (47.5)	31 (52.5)	0.281
≥50 years	41 (41)	15 (36.6)	26 (63.4)	-
Menopausal status, n (%)				
Premenopausal	65 (65)	31 (47.7)	34 (52.3)	0.199
Postmenopausal	35 (35)	12 (34.3)	23 (65.7)	-
Height (cm)				
Median (range)	164 (141–181)	163 (141–181)	164 (152–176)	0.63
Weight (kg)				
Median (range)	62 (42–98)	62 (42–98)	63 (46–92)	0.44
BMI, n (%)				
Normal <25	68 (68)	31 (45.6)	37 (54.4)	0.447
Overweight / obesity	32 (32)	12 (37.5)	20 (62.5)	-
MBD, n (%)				
A-B	17 (17)	8 (47.1)	9 (52.9)	0.731
C-D	80 (80)	34 (42.5)	46 (57.5)	-
Missing	3 (3)	1 (33.3)	2 (66.7)	
Cancer subtype, n (%)				
HER2+	35 (35)	20 (57.1)	15 (42.9)	0.01
Triple negative	31 (31)	17 (54.8)	14 (45.2)	0.03
Luminal	34 (34)	6 (17.6)	28 (82.4)	-
Histological grade, n (%)				
G2	41 (41)	16 (39)	25 (61)	0.198
G3	55 (55)	27 (49.1)	28 (50.9)	-
Gx	4 (4)	0 (0)	4 (100)	
T-score, n (%)				
T1-2	83 (83)	37 (44.6)	46 (55.4)	0.915
T3-4	13 (13)	6 (46.2)	7 (53.8)	
Tx	4 (4)	0 (0)	4 (100)	
Nodal status, n (%)				
N0	50 (50)	23 (46)	27 (54)	0.733
N1	47 (47)	20 (42.6)	27 (57.4)	-
Nx	3 (3)	0 (0)	3 (100)	
Ob-R cell positivity, n (%)				
≤50%	48 (48)	13 (27.1)	35 (72.9)	0.002
>50%	52 (52)	30 (57.7)	22 (42.3)	-

* We have calculated the column percentage for all patients. We have calculated the row percentages for patients with and without pCR. The bold p-values denote statistical significance at 5% alpha level. Abbreviations: BMI, body mass index; G, histological grade; HER2, human epidermal growth factor receptor 2; HR, hormonal receptor; MBD, mammographic breast density; N, nodal staging score; Ob-R, leptin receptor; pCR, pathological complete response; T, tumor score. Missing, Gx, Tx, and Nx values have been excluded from the analyses. n: number of samples

**Table 2:** Ob-R cell overexpression (>50%) in accordance with baseline characteristics: univariate and multivariate logistic regression model analyses. (**A**)Univariate analyses;(**B**)Multivariate analyses.

Outcome: Ob-R > 50%	(A) Univariate Analyses					
	OR	95%CI	*p*-Value			
Patients with < 50 y	2.9	1.3 to 6.67	0.011			
Premenopausal status	3.03	1.32 to 6.67	0.011			
Patients with BMI > 25	1.88	0.8 to 4.5	0.152			
Cancer type						
HER2+ vs. Luminal	3.10	1.18 to 8.52	0.024			
Triple negative vs. Luminal	2.54	0.94 to 7.08	0.068			
**Outcome: Ob-R > 50%**	**(B) Multivariate Analyses**					
	**Menopausal Status as Factor**			**Age as Factor**		
	**OR**	**95%CI**	***p*-Value**	**OR**	**95%CI**	***p*-Value**
Patients < 50 y	-	-	-	5	2 to 14.3	0.001
Premenopausal status	5	2 to 14.3	0.001	-	-	-
BMI > 25	3.52	1.35 to 11.88	0.017	3.50	1.27 to 10.54	0.019
Cancer type						
HER2+ vs. Luminal	5.91	0.12 to 3.13	0.002	6.82	2.18 to 24.21	0.002
TNBC vs. Luminal	2.87	0.07 to 1.71	0.058	2.66	0.9 to 8.08	0.077

The univariate and multivariate analyses have been conducted with logistic regression models. The multivariate analyses included all prognostic factors reported in Table 1 in the full model. Factors included in the final model were selected at a *p*-value of <0.1 or change in OR > 10% compared to the full model. The factors selected in the final model are presented. Ob-R overexpression was greater in patients aged < 50 years, who were premenopausal, had a BMI ≥ 25 kg/m^2^, or who were HER2 or TNBC positive compared with those aged ≥ 50 years, postmenopausal, with normal weight, or who had the luminal subtype.The bold p-values denote statistical significance at 5% alpha level. BMI: Body mass index, HER2: Human epidermal growth factor receptor 2, HR: Hormonal receptor, Ob-R: Leptin receptor.

## Data Availability

The datasets generated and/or analyzed during the current study are available from the corresponding author on reasonable request.
